# Structure and Stability of Aromatic Nitrogen Heterocycles Used in the Field of Energetic Materials

**DOI:** 10.3390/molecules25143232

**Published:** 2020-07-15

**Authors:** He-Hou Zong, Chuang Yao, Chang Q Sun, Jian-Guo Zhang, Lei Zhang

**Affiliations:** 1 Institute of Chemical Materials, China Academy of Engineering Physics (CAEP), Mianyang 621900, China; zongzbobo@163.com; 2 CAEP Software Center for High Performance Numerical Simulation, Beijing 100088, China; 3 Key Laboratory of Extraordinary Bond Engineering and Advance Materials Technology (EBEAM) of Chongqing, School of Materials Science and Engineering, Yangtze Normal University, Chongqing 408100, China; yaochuang@yznu.cn (C.Y.); ecqsun@ntu.edu.sg (C.Q.S.); 4 State Key Laboratory of Explosion Science and Technology, Beijing Institute of Technology, Beijing 100081, China; 5 Laboratory of Computational Physics, Institute of Applied Physics and Computational Mathematics, Beijing 100088, China

**Keywords:** nitrogen heterocycles, nitrogen lone pair, electron delocalization, σ-aromaticity

## Abstract

Understanding the stabilization of nitrogen heterocycles is critical in the field of energetic materials and calls for innovative knowledge of nitrogen aromatics. Herewith, we report for the first time that nitrogen lone pair electron (NLPE) delocalization in five-membered nitrogen heterocycles creates a second σ-aromaticity in addition to the prototypical π-aromaticity. The NLPE delocalization and the attendant dual-aromaticity are enhanced as more carbon atoms in the ring are substituted by unsaturated nitrogen atoms. The presence of adjacent nitrogen atoms in the ring can enhance the aromaticity of the nitrogen heterocycles and improve in-crystal intermolecular binding strength but will decrease the firmness of the individual molecular architecture. Notably, such σ-aromaticity is not present in six-membered nitrogen heterocycles, probably due to the longer bonds and broader regions of their rings; therefore, six-membered heterocycles present overall lower aromaticity than five-membered heterocycles. This work brings new knowledge to nitrogen aromatics and is expected to inspire broad interest in the chemistry community.

## 1. Introduction

Compared to organic chemistry, the development of nitrogen chemistry has been very slow since the Haber–Bosch process was invented in the early 1900s to produce ammonia industrially from nitrogen [1]. A typical example is the full-nitrogen substance pentazole anion *cyclo*-N_5_^−^, which has super-high energy storage in its N−N bond and is considered the ultimate pursuit of energetic materials. The *cyclo*-N_5_^−^, albeit known to be π-aromatic like benzene, exhibits completely opposite properties to benzene—it is surprisingly unstable, difficult to obtain, and susceptible to electrophilic attack. After the first failed attempt in 1903 [2], the *cyclo*-N_5_^−^ was finally isolated in a stable crystal after 114 years of continuous effort [3]. However, the mechanism of the stabilization of *cyclo*-N_5_^−^ and the means of achieving productive separation of *cyclo*-N_5_^−^ need further exploration [4,5].

Coincidentally, anomalous phenomena also occur in the aza systems of alicyclic amines, which are an important composition of energetic materials [6]. According to valence shell electron pair repulsion (VSEPR) theory, the nitrogen lone pair electrons (NLPEs) in a cyclic system should repel the neighboring C-N bonds and other lone pairs in the ring [7,8,9], thereby destabilizing the cyclic aza system. However, the presence of nitrogen in the ring has been proved to confer more degrees of stability to these molecules than their hydrocarbon analogues [6,7], suggesting the failure of VSEPR theory in interpreting the stabilization of the cyclic aza species. Some researchers have proposed that delocalization of the nitrogen lone pair (n→σ*) in the cyclic aza systems accounts for the enhancement of the stability of the cycles [6,10]. However, better understanding of the “aza-stabilization” anomaly calls for new knowledge of nitrogen chemistry. 

Herewith, we report a series of quantum calculations on the structure, molecular orbital, electron density, magnetic shielding, and energetics of dozens of compounds composed of various homocycles and nitrogen heterocycles. We discovered for the first time the presence of a second σ-aromaticity in five-membered nitrogen heterocycles, in addition to the prototypical π-aromaticity; however, such σ-aromaticity is not present in six-membered nitrogen heterocycles. This work presents the key role of NLPEs in distinguishing the chemical properties of nitrogen aromatics from those of carbon aromatics and is expected to inspire broad interest in nitrogen chemistry. 

## 2. Results and Discussion

### 2.1. Enhanced Aromaticity of Nitrogen Heterocycles Due to NLPE Delocalization

We collected 22 types of molecules, with increasing carbon atoms substituted by nitrogen atoms (see Table 1). All the molecular structures were first optimized by energy minimization and then used for visualization of molecular orbital (MO) and analysis of charge density, magnetic shielding, and bond characteristics. Notably, after geometry optimization, **14** presented slight distortion from the planar conformation (by 0.18 Å), and all the other cyclic molecules remained planar. 

#### 2.1.1. Visualization of the NLPE Delocalization

The nitrogen in **1** (ammonia) was sp^3^ hybridized, and the carbon and nitrogen in all the cyclic compounds (**2**–**22**) were sp^2^ hybridized. The σ NLPE delocalization, as well as the π electron delocalization of the 22 systems, was visualized by the isosurfaces of each lowest molecular orbital (MO), as shown in Table 1. The quantities with min-π/min-σ(LP) subscripts were the components contributed by the lowest energy level of π-electrons/σ-NLPEs.

We first studied the cases with a single unsaturated nitrogen atom (**1**, **3**, **16**, and **17**), with each of these systems presenting only one pair of σ-NLPEs. For example, ammonia (**1**) has one pair of NLPEs at the top of the trigonal pyramidal of the structure; **3** has one pair of NLPEs in the ring; for **16** and **17**, the NLPEs of the saturated nitrogen join the lower-energy π MO, so there is only one pair of σ-NLPEs affiliated with the unsaturated nitrogen in each heterocycle. As shown by the shapes of their **MO_min-σ(LP)_** in Table 1, these σ-NLPEs were highly localized in the region of the unsaturated nitrogen atom. The high localization of the σ-NLPEs makes these systems basic and attractive to electrophiles, which is consistent with traditional organic/inorganic chemistry knowledge [7]. 

However, the presence of more than two unsaturated nitrogen atoms in the ring, partially in the ortho positions, made the NLPEs delocalized in a broader region. For example, **4**, **7**, **10**–**12**, and **18**–**21** had the isosurfaces of their **MO_min-σ(LP)_** continuously distributed in the region between the unsaturated nitrogen atoms, as shown in Table 1. Notably, although **8** and **13** had adjacent unsaturated nitrogen atoms in the ring, some of these nitrogen atoms contributed their NLPEs to the relative lower energy π orbital instead of the σ orbital. Therefore, these two compounds did not have ortho-NLPEs, but showed para- and para-NLPEs in each **MO_min-σ(LP)_**. Table 1 indicates that the σ-NLPEs in **5**, **6**, **8**, **9**, and **13**, which had para or meta position in each ring, were generally more localized compared to those NLPEs in the ortho positions.

In extreme cases, when all carbon atoms were substituted by unsaturated nitrogen atoms in the ring, the delocalization of the NLPEs formed into a circle and achieved a maximum. For example, the **MO_min-σ(LP)_** of **14** and **22** spread out over each molecule into a flower shape in the equatorial plane, as shown in Table 1. This is consistent with our previous study on pentazolate anion [11,12]. 

In brief summary, the substitution of carbon atoms by unsaturated nitrogen atoms in the heterocycles improved the extent of the delocalization of NLPEs. More unsaturated nitrogen atoms led to a higher extent of NLPE delocalization, and the preferable order of the NLPE positions that contributed to such delocalization was ortho > meta > para.

#### 2.1.2. Quantification of the NLPE Delocalization

**Figure 1:**
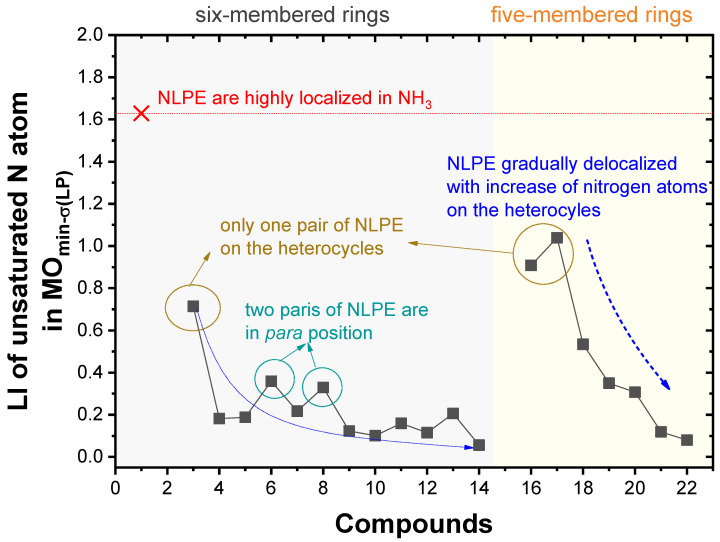
Localization index (LI) of unsaturated nitrogen atom in each lowest σ(LP) orbital for the 22 studied compounds.

In order to quantify the delocalization extent of the NLPEs, we calculated the electron-based aromaticity LI [13,14]. The LI quantitatively measured how many electrons were localized in a region; a higher LI value suggested stronger localization, but weaker delocalization of the NLPEs. The LI of the unsaturated nitrogen atoms projected in **MO_min-σ(LP)_** for each of the 22 studied compounds is shown in Figure 1.

Figure 1 confirms that, in the systems with a single unsaturated nitrogen atom, such as **1** (LI = 1.63), **3** (LI = 0.71), **16** (LI = 0.91), and **17** (LI = 1.04), the NLPEs were highly localized. The NLPEs in the para positions (LI = 0.36 for **6** and LI = 0.33 for **8**, with 2 pairs of NLPEs in the para positions) contributed less to the delocalization of the heterocycles, compared to those NLPEs in the ortho and meta positions (LI = 0.18 for **4**, with 2 pairs of NLPEs in the ortho position; LI = 0.19 for **5**, with 2 pairs of NLPEs in the meta position). 

The extent of the NLPE delocalization gradually increased as a function of the number of unsaturated nitrogen atoms in the ring. Notably, the change of the NLPE delocalization in the five-membered heterocycles (with LI varying in 1.04–0.08) was more dramatic than in the six-membered heterocycles (with LI varying in 0.71–0.06).

#### 2.1.3. Discovery of Additional σ-Aromaticity in Five-Membered Nitrogen Heterocycles

To determine how aromaticity (including prototypical π-aromaticity, newly proposed σ-aromaticity, and total aromaticity) varies in these heterocycles, we further calculated the magnetic index of aromaticity–NICS_zz_(r), which is the NICS value along the z axis, by far the most widely used method for diagnosing aromaticity [15,16]. The indices with π/σ subscripts were the components contributed by all the π-electrons/σ-electrons. The more negative the NICS values, the more aromatic were the rings.

**Figure 2:**
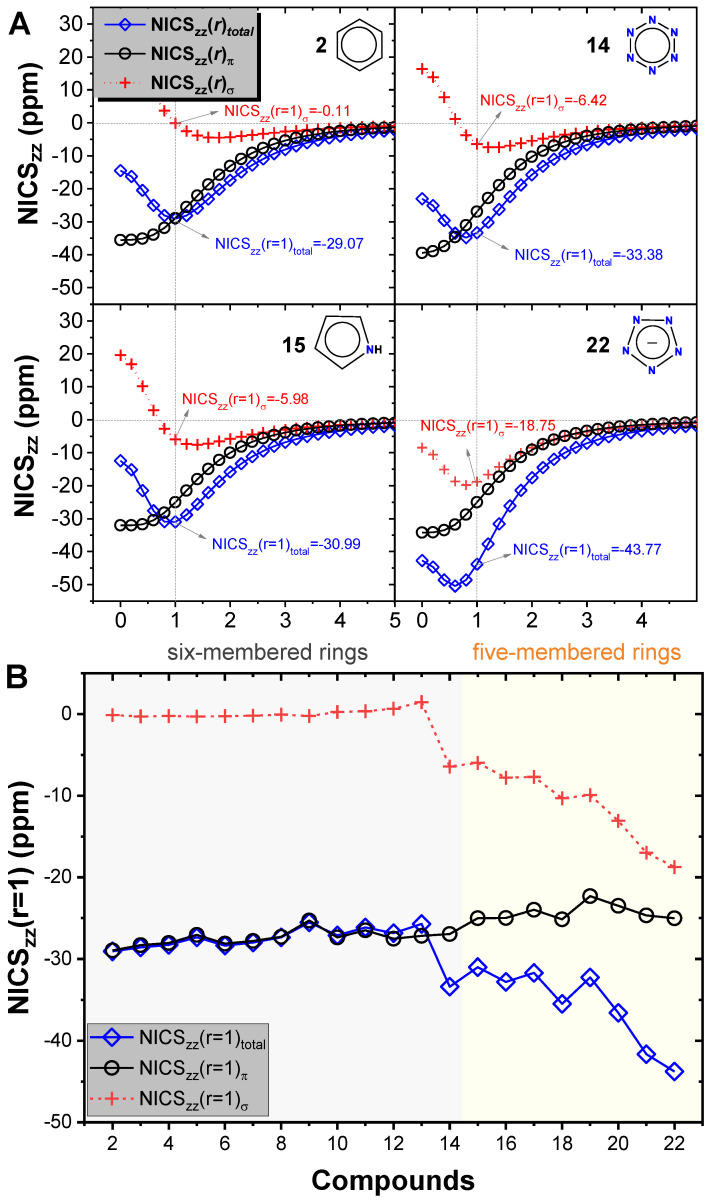
(**A**) NICS_zz_(*r*)_total_, NICS_zz_(*r*)_π_, and NICS_zz_(*r*)_σ_ of **2**, **14**, **15**, and **22** as a function of *r*, the vertical distance relative to the ring critical point. (**B**) NICS_zz_(*r* = 1)_total_, NICS_zz_(*r* = 1)_π_, and NICS_zz_(*r* = 1)_σ_ of all 21 of the studied cyclic systems.

Taking **2**, **14**, **15**, and **22** as examples, we showed the NICS_zz_(*r*)_total_, as well as the π and σ orbital components (NICS_zz_(*r*)_π_ and NICS_zz_(*r*)_σ_) in Figure 2A. As the vertical distance relative to the ring critical point varied from *r* = 0.0 to 5.0 Å, NICS_zz_(*r*)_π_ was always negative for **2**, **14**, **15**, and **22**, suggesting the presence of π-aromaticity in these systems. NICS_zz_(*r*)_σ_ was always negative in **22**, whereas it was positive or close to zero in **2**; the situations of **14** and **15** were between **2** and **22**. This indicated the presence of σ-aromaticity in the all-nitrogen compound **22**, whereas benzene **2** lacked such σ-aromaticity; **14** and **15** had only weak σ-aromaticity. 

The order of the σ-aromaticity of the four compounds could be quantified by the absolute values of NICS_zz_(1)_σ_: **22** > **14** > **15** > **2**. Here NICS_zz_(1) was the NICS_zz_ value when the vertical distance relative to the ring critical point was 1 Å. Due to the significant contribution of σ-aromaticity in the four compounds, their overall aromaticity presented an identical order to the σ-aromaticity, with the order of the absolute values of NICS_zz_(1)_total_ being **22** > **14** > **15** > **2**.

We further performed NICS_zz_(*r*)_total_, NICS_zz_(*r*)_π_, and NICS_zz_(*r*)_σ_ calculations for all other cyclic systems when *r* varied from 0.0 to 5.0 Å. The highest absolute value of the NICS_zz_(*r*)_total_ for **13** and **15** was *r*_extreme_ = 1.0 Å vertically above the ring critical point; for **14** and **16**–**20**, *r*_extreme_ = 0.8 Å; for **21** and **22**, *r*_extreme_ shifted to 0.6 Å, identical to the P_2_N_3_^−^ anion [17]. In order to evaluate the aromaticity of all 21 cyclic systems in identical conditions, we uniformly took the NICS_zz_(*r*) values at *r* = 1 Å to compare their aromaticity, as shown in Figure 2B. 

**Figure 3:**
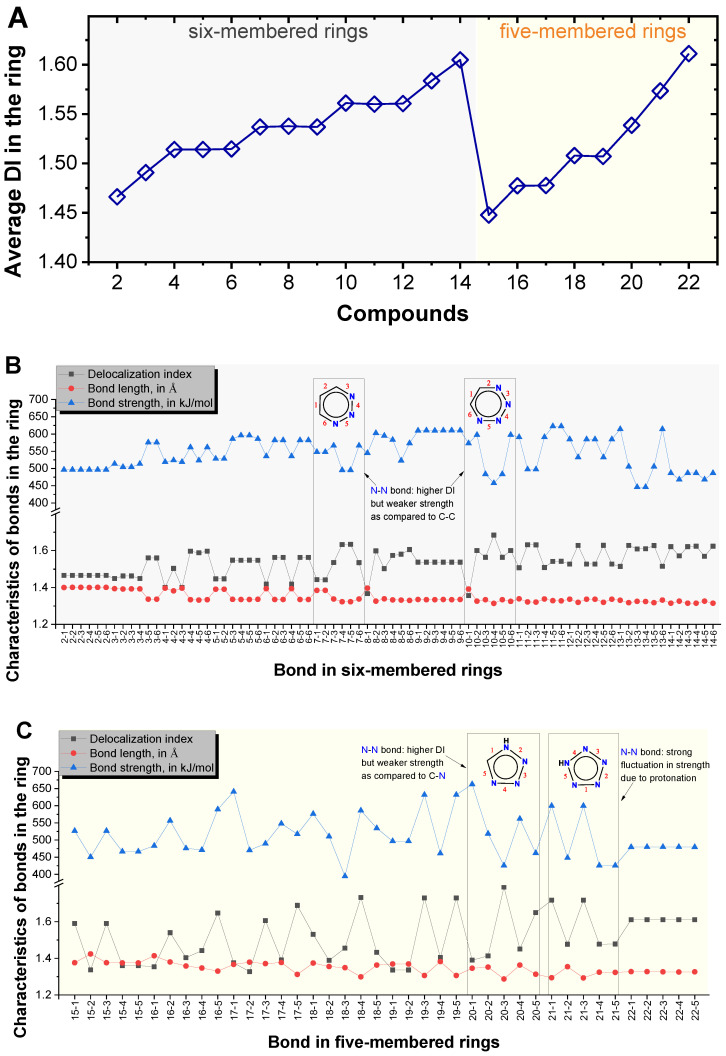
(**A**) Average delocalization index (DI) of the bonds in the ring for the 21 cyclic compounds. (**B**) DI, length, and strength for individual bonds in the rings of the six-membered compounds and (**C**) five-membered compounds. X-Y in *x*-axis denotes bond Y of compound X; the Y bond indices for each compound are illustrated in Table 1.

Figure 2B clearly indicates that σ-aromaticity was present in all five-membered rings, and it was gradually enhanced as the number of nitrogen atoms in the ring increased; σ-aromaticity reached a maximum in **22**, in which all carbon atoms were substituted by unsaturated nitrogen atoms. In addition, we found a significant influence of bond length in the ring on the aromaticity of the compound; longer bonds in the ring led to weaker aromaticity. For example, **16** and **17** were isomers; the latter had longer bonds in the ring (Figure 3) and thereby had smaller π-aromaticity, σ-aromaticity, and total aromaticity. Similar phenomena occurred also in isomeric **18** and **19**. Interestingly, except for **14**, no obvious σ-aromaticity was present in the six-membered rings, probably due to the longer bonds and broader regions of their rings compared to the five-membered rings. 

Due to the contribution of the NLPE delocalization, the five-membered rings showed dual-aromaticity, namely π- and σ-aromaticity. Figure 2B indicates that dual-aromaticity of the five-membered rings, with NICS_zz_(1)_total_ in the range of −43.77 to −30.99, was significantly higher than the prototypical aromaticity of the six-membered rings, which had their NICS_zz_(1)_total_ in the range of −29.07 to −25.53. 

We note that the dual aromaticity in five-membered nitrogen heterocycles is very different from the double aromaticity of metallic compounds. The dual aromaticity of, for example, Al_4_^2−^ dianion means that four σ electrons and two π electrons together form a single aromatic system due to the electron deficiency of the dianion [18,19,20]. However, the dual aromaticity of five-membered nitrogen heterocycles was derived from two separate aromatic systems, with independently delocalized π-electrons and σ(LP)-electrons, like those present in 3,5-dehydrophenyl cation [21], saturated inorganic rings [22], and probably in pnictogen five-membered rings like P_5_^−^ and As_5_^−^ anion [23,24].

Therefore, the “dual” aromaticity in five-membered nitrogen heterocycles means two types of electrons and two separate aromatic systems. As we declared in one of our previous works, the two aromatic systems are independent in real space but are coupled in energy space. The competition between the nonbonding interactions in both aromatic systems and the LP–LP repulsive interactions in the σ aromatic system makes the dual-aromatics show different reactivity to electrophilic attack in different acidic solutions [11].

### 2.2. Effect of Enhanced Aromaticity of Heterocycles on Structure Stability

In this section, we investigate the effect of enhanced aromaticity of nitrogen heterocycles on their structure stability, at both the molecular and crystal levels. Notably, the calculation method for crystal systems considers periodic boundary condition and intermolecular interactions and thereby can reflect the effect of aromaticity on the energetics of solid-state systems.

#### 2.2.1. Reduced Molecular Structure Firmness

Structure stability of nitrogen heterocycles is closely related to the firmness of their backbone bonds. Herewith, we quantified the firmness of the bonds in the ring by their DI, bond length, and bond strength, as shown in Figure 3.

DI [14], also called fuzzy bond order [25,26], measures the number of shared electrons between two atoms. Figure 3A clearly indicates that the average DI of the ring, namely the electron delocalization over the cyclic backbone, was enhanced as the number of unsaturated nitrogen atoms increased. 

The characteristics of each individual bond in the rings of the 21 cyclic compounds, including DI, length, and strength, are shown in Figure 3B,C. Bond length was calculated using the HASEM application [11,27]. Figure 3 indicates that higher DI and smaller bond length generally corresponded to higher bond strength. However, we found that N−N bonds presented significantly lower strength as compared to C−C or C−N bonds, even when they had higher bond orders (applicable when DI ≤ 2); this finding is consistent with a previous report [28]. 

When more carbons are substituted by nitrogen atoms, the attendant phenomenon is that more N−N bonds, which have low strength and are very likely to rupture, will be present. Therefore, although NLPEs are more delocalized in the ring and even create additional σ-aromaticity, the firmness of the entire molecular architecture is reduced once adjacent nitrogen atoms are present in the ring. For example, bond 4 and 5 in compound **7** (7-4 and 7-5) in Figure 3B; bond 4 in compound **10** (10-4) in Figure 3B; bond 3 in compound **20** (20-3) and so on. These mentioned N–N bonds have much lower strength compared to C−C or C−N bonds in the rings. 

The very low N-N bond strength of *cyclo*-pentazolate anion (**22**) is the reason that **22** is difficult to productively separate, because these N-N bonds easily break prior to the C−N bond cleavage in the precursor 3,5-dimethyl-4-hydroxyphenylpentazole [3]. One practical way, as reported in our recent work [11], is to introduce an appropriate concentration of hydronium or ammonium in the solution, which strengthens all the N–N bonds in **22** with the aid of the electron delocalization of the formed hydrogen bonds.

#### 2.2.2. Improved In-Crystal Intermolecular Binding Strength

Crystal structure stability depends significantly on the interspecies binding strength of the constitution components. Therefore, the stability of the crystals composed of nitrogen heterocycles is closely related to one of the important intermolecular–aromatic interactions. 

**Figure 4:**
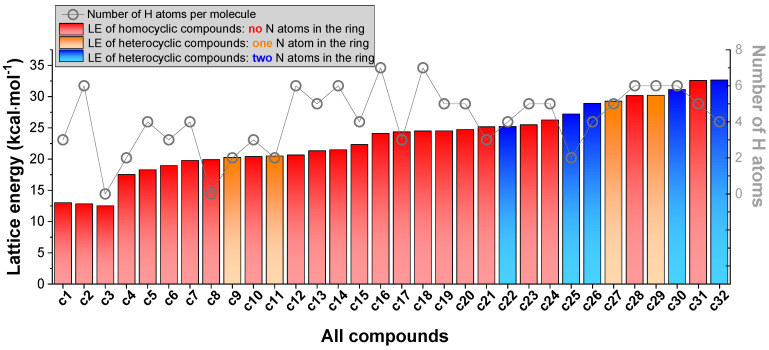
Lattice energy (LE) of 32 crystals composed of ammonia (red bar), homocycles (red bar), and heterocycles (with one N in the ring, orange bar; two N in the ring, blue bar). Number of hydrogen atoms in each molecule is plotted by grey open circles.

Herewith, we collected 32 crystal structures composed of ammonia and various homocyclic and nitrogen heterocyclic molecules (Table 2). Lattice energy (LE), the energy difference between total energy of constituent molecules in the free state and total energy of the crystal, was employed to quantify the in-crystal binding strength, as shown in Figure 4. The number of hydrogen atoms in each molecule was also plotted to evaluate the effect of hydrogen bonding on LE. 

As shown in Figure 4, **c1** (ammonia), which has no aromaticity, is gaseous at ambient condition and has the lowest LE of 13.01 kcal·mol^−1^. As for the cyclic systems, Figure 4 indicates that the crystals composed of C–N heterocycles generally have higher stability as compared to those composed of all-C homocyclic molecules. One typical example is **c9** and **c11**, which have one nitrogen atom in each heterocycle. Both have relatively less hydrogen bonding than **c5**–**c7**, but present higher LE (20.27 and 20.49 kcal·mol^−1^ for **c9** and **c11**, respectively) than **c5**–**c7** (LE in the range of 18.28–19.78 kcal·mol^−1^). Another typical example is **c25** and **c32**, which have two nitrogen atoms in each heterocycle. Both have much less hydrogen bonding than **c23** and **c24**, whereas they present higher LE (27.22 kcal·mol^−1^ for **c25** and 32.67 kcal·mol^−1^ for **c32**, respectively) due to their relatively stronger aromaticity. In addition, the presence of nitro group is conducive to provoking dipole–dipole interactions, thereby further enlarging LE of the crystal. 

**c2**–**c8**, on the other hand, have no nitrogen atoms in their rings, relatively less hydrogen bonding, and present very low LE (in the range of 12.83–19.91 kcal·mol^−1^, respectively). For example, **c2** (benzene) has very low LE (12.83 kcal·mol^−1^) and is liquid at ambient condition. **c3** (HNB explosive), with a low LE = 12.50 kcal·mol^−1^, is very sensitive to light and easy to decompose. **c12**–**c21** generally have abundant hydrogen bonding, whereas they have relatively lower LE than the crystals composed of heterocyclic molecules (like **c22**, **c25**, **c26**, **c27**, **c29**, **c30**, and **c32**) due to the absence of nitrogen atoms in their rings and therefore relatively weaker aromaticity. 

In brief, we found that strong aromaticity of heterocyclic molecules is conducive to enhancing in-crystal intermolecular binding, and vice versa.

## 3. Computational Methodology

In order to study NLPE delocalization characteristics and the attendant changes in aromaticity and structure stability of nitrogen heterocycles, we collected 22 molecular structures and 32 crystal structures composed of ammonia and six- and five-membered homocycles and nitrogen heterocycles. All of these are functional groups or molecules of commonly seen energetic materials.

At the molecular level, the structure optimization was performed using Gaussian 09 at the B3LYP/6-311++G(d,p) level of theory [29]. The optimized structures were then used for charge density analysis. All the electron-based aromaticity indices, namely the localization index (LI) and the delocalization index (DI) [14,25,26], were calculated at the CCSD(T)/6-31G+ level, and the magnetic based aromaticity index (nucleus-independent chemical shifts, NICS [15,16]) were calculated at the B3LYP/6-311++G(d, p) level. Using natural bond orbital (NBO) analysis and the quantum theory of atoms in molecules (QTAIM) method, the Multiwfn application [13] was used to separate DI and NICS into individual components contributed solely by specified orbitals. 

The crystal level calculations were performed using High Accuracy Atomistic Simulation for Energetic Materials (HASEM) software [27,30], with the inputs taken from the lattice parameters and atomic coordinates of the CIF files stored in the Cambridge Crystallographic Data Centre (CCDC) database [31]. All the geometry optimization was performed using the conjugate gradient method. The simulated structures were considered to be optimized when the residual forces were less than 0.03 eV/Å, and the stress components were less than 0.01 GPa. The optimized structures were then used for energetics evaluation.

## 4. Conclusions

We performed a quantum a chemistry study on 54 aza systems (22 molecules and 32 crystals) and studied the aromaticity of various nitrogen heterocycles from the perspectives of structure, molecular orbital, electron density, magnetic shielding, and energetics. The main conclusions are as follows:

(1) More unsaturated nitrogen atoms present in the heterocycles lead to a higher extent of nitrogen lone pair electrons (NLPE) delocalization, and the preferable order of the NLPE position that contributes to such delocalization is ortho > meta > para.
(2) σ-aromaticity is discovered in five-membered nitrogen heterocycles, in addition to the prototypical π-aromaticity; however, such σ-aromaticity is not present in six-membered nitrogen heterocycles.
(3) The presence of adjacent nitrogen atoms in the ring of nitrogen heterocycles (ortho) decreases the firmness of the molecular structure, but can increase the in-crystal intermolecular binding strength.

## Figures and Tables

**Table 1:** Chemical structures of ammonia (**1**), benzene (**2**), six-membered nitrogen heterocycles (**3**–**14**), and five-membered nitrogen heterocycles (**15**–**22**). The isosurfaces (0.05) of the lowest σ MO containing nitrogen lone pair electrons (**MO_min-σ(LP)_**) and the lowest π MO (**MO_min-π_**) are presented for each of the 22 compounds.

**Label**	**1**	**2**	**3**	**4**	**5**	**6**
**Structure**	NH_3_	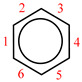	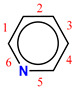	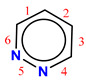	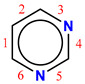	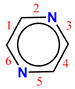
**MO_min-σ(LP)_**	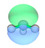	-	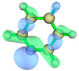	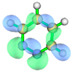	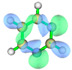	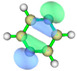
**MO_min-π_**	-	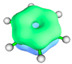	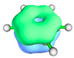	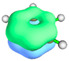	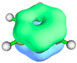	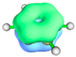
**Label**	**7**	**8**	**9**	**10**	**11**	**12**
**Structure**	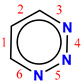	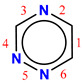	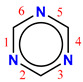	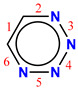	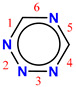	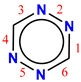
**MO_min-σ(LP)_**	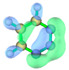	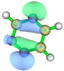	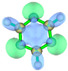	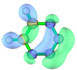	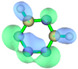	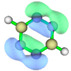
**MO_min-π_**	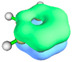	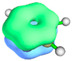	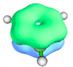	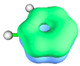	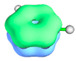	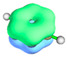
**Label**	**13**	**14**	**15**	**16**	**17**	**18**
**Structure**	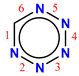	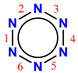	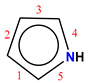	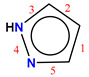	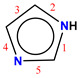	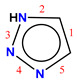
**MO_min-σ(LP)_**	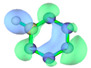	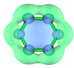	-	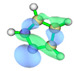	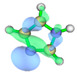	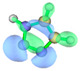
**MO_min-π_**	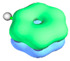	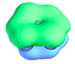	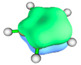	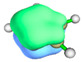	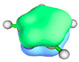	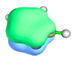
**Label**	**19**	**20**	**21**	**22**		
**Structure**	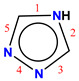	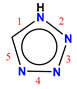	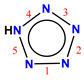	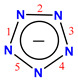		
**MO_min-σ(LP)_**	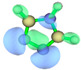	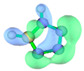	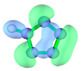	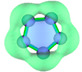		
**MO_min-π_**	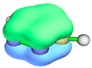	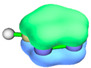	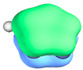	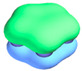		

**Table 2:** Lattice energies (LE, in kcal·mol^−1^) of 32 crystals composed of ammine and various nitrogen heterocycles. Cambridge Crystallographic Data Centre (CCDC) number of the crystal structures, as well as the chemical structures of the constituent molecules are provided.

Label	CCDC No.	Structure	LE	Label	CCDC No.	Structure	LE
**c1**	1644462	NH_3_	13.01	**c17**	1272845	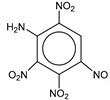	24.35
**c2**	725244	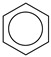	12.83	**c18**	1272852	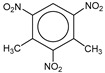	24.49
**c3**	1177301	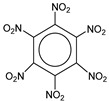	12.50	**c19**	1214795	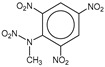	24.53
**c4**	1166481	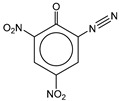	17.52	**c20**	125117	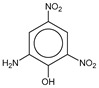	24.73
**c5**	1142965	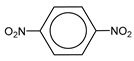	18.28	**c21**	1255528	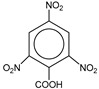	25.17
**c6**	213311	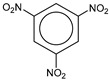	18.97	**c22**	1910475	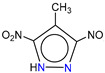	25.22
**c7**	201615	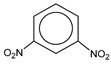	19.78	**c23**	1105564	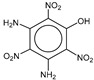	25.50
**c8**	171054	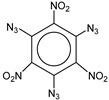	19.91	**c24**	1136625	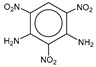	26.26
**c9**	258373	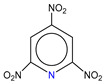	20.27	**c25**	166510	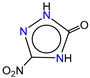	27.22
**c10**	667816	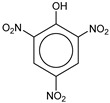	20.40	**c26**	938305	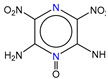	28.93
**c11**	258373	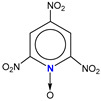	20.49	**c27**	910889	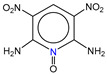	29.28
**c12**	225824	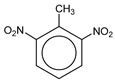	20.65	**c28**	1266837	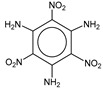	30.17
**c13**	227799	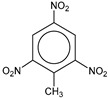	21.32	**c29**	1270764	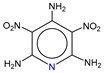	30.22
**c14**	225823	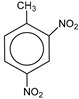	21.48	**c30**	273637	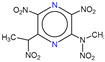	31.14
**c15**	947035	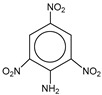	22.32	**c31**	1135164	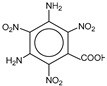	32.58
**c16**	1272856	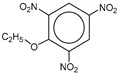	24.13	**c32**	938305	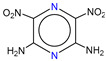	32.67
